# *In vivo* Screen Identifies *Zdhhc2* as a Critical Regulator of Germinal Center B Cell Differentiation

**DOI:** 10.3389/fimmu.2020.01025

**Published:** 2020-06-10

**Authors:** Rongqing Zhao, Huihui Zhang, Yan Zhang, Dan Li, Chuanxin Huang, Fubin Li

**Affiliations:** ^1^Shanghai Institute of Immunology, Faculty of Basic Medicine, Shanghai Institute of Immunology, Shanghai Jiao Tong University School of Medicine, Shanghai, China; ^2^Boston Consulting Group, Shenzhen, China; ^3^Key Laboratory of Cell Differentiation and Apoptosis of Chinese Ministry of Education, Shanghai Jiao Tong University School of Medicine, Shanghai, China; ^4^Collaborative Innovation Center of Systems Biomedicine, Shanghai Jiao Tong University, Shanghai, China

**Keywords:** germinal center B cell, *in vivo* screen, shRNA, GC selection, *Zdhhc2*

## Abstract

Germinal center (GC) B cell differentiation is critical for the production of affinity-matured pathogen-specific antibodies, the dysregulation of which may lead to humoral immunodeficiency or autoimmunity. The development of an *in vivo* screening system for factors regulating GC B cell differentiation has been a challenge. Here we describe a small-scale *in vivo* screening system with NP-specific B1-8^hi^ cells and a retroviral shRNA library targeting 78 candidate genes to search for B cell-intrinsic factors that specifically regulate GC B cell differentiation. *Zdhhc2*, a gene encoding palmitoyltransferase ZDHHC2 and highly expressed in GC B cells, is identified as a strong positive regulator of GC B cell differentiation. B1-8^hi^ cells transduced with *Zdhhc2*-shRNA are severely compromised in differentiating into GC B cells. A further analysis of *in vitro* differentiated B cells transduced with *Zdhhc2*-shRNA shows that *Zdhhc2* is critical for the proliferation and the survival of B cells stimulated by CD40L, BAFF, and IL-21 and consequently impacts on their differentiation into GC B cells and post-GC B cells. These studies not only identify *Zdhhc2* as a novel regulator of GC B cell differentiation but also represent a proof of concept of *in vivo* screen for regulators of GC B cell differentiation.

## Introduction

The germinal center (GC) B cell response is a fundamental process in humoral immunity that enables the rapid evolution and selection of B cells, so B cells that express pathogen-specific affinity-matured B cell receptors (BCRs) with reduced autoreactivity can be selected for antibody production (1, 2). GC B cell differentiation is a T cell-dependent process in which antigen-specific B cells present antigens to cognate T cells, which in turn provide reciprocal activating signals for the differentiation for both GC B cells and follicular T helper (T_FH_) cells. Antigen-specific B/T cell interactions result in the activation and the proliferation of both B and T cells and their chemokine-dependent migration to the B cell follicles to form the GCs (3–7). In the dark zone of GCs, B cells proliferate rapidly and undergo extensive activation-induced cytidine deaminase-mediated somatic hypermutation, which leads to BCR diversification (8). The expanded and BCR-diversified B cells migrate to the light zone of GCs and compete for capturing antigens and T cell help (6, 9–13). It has been proposed that high-affinity B cells can capture and present more antigens to T_FH_ cells and consequently acquire sufficient signals from T_FH_ cells for their further differentiation into plasmablasts and memory B cells (14–16). At the same time, autoreactive B cells are counter-selected to ensure the production of pathogen-specific affinity-matured BCRs with reduced autoreactivity (2). The integrity of this process is critical for humoral immunity and requires careful regulation (17). A deficiency in the key signals involved in this process, such as CD40L/CD40 and ICOSL/ICOS signals, can lead to either humoral immune deficiency or autoimmune diseases, or sometimes both, such as lupus (18–21). Therefore, it is crucial to understand the molecular mechanism that regulates GC B cell differentiation.

Previously, a number of factors critical for GC B cell differentiation have been identified (22–27), but none of them was initially identified through *in vivo* screen, as far as we know. For instance, BCL6 (encoded by *Bcl6*), a key transcription factor essential for GC B cell differentiation, was noticed because of its mutations in GC B cell-derived diffuse large B cell lymphoma (22). *Bcl6*-deficient mice have normal primary follicles but lack GCs after immunization (22, 28). MYC, another important transcription factor essential for GC formation and maintenance (29), was initially noticed due to its upregulated expression in B cell-derived Burkitt lymphoma (30). IRF4, critical for GC formation and antibody production (24, 25), was initially cloned from a translocation breakpoint in myeloma (31). More recently, several factors (such as ephrin B1 and Cbl ubiquitin ligases) involved in regulating GC B cell differentiation were identified due to their distinct expression patterns in GC B cells (32, 33). The development of *in vivo* screening systems for B cell-intrinsic factors regulating GC B cell differentiation has been a challenge, which has hindered the discovery of new genes implicated in GC B cell differentiation.

*In vivo* screens in mouse models have been mainly applied in the context of tumorigenesis based on either spontaneous or site-directed mutagenesis approaches, such as mutation-inducing chemicals, shRNA, and CRISPR/Cas9 systems (34–47). These screens are based on the principles that either gain-of-function mutations in oncogenes or loss-of-function mutations in tumor-suppressive genes can promote tumorigenesis in various tumor models, including tumors derived from B- and T-lineage cells, breast cancer, and glioblastoma (34, 35, 37, 44). A similar strategy has also been exploited to screen genes that regulate B cell differentiation in the bone marrow, where both positive and negative selections take place (48). In a screen for microRNA that regulates B cell tolerance, miR-148a was identified as a critical regulator of B cell tolerance and autoimmunity that can promote the survival of autoreactive immature B cells (48). In another screen for genes that regulate T cell differentiation during lymphocytic choriomeningitis virus infection, *Ccnt1* was identified to promote both CD4 and CD8 T cell differentiation (49).

Since the *in vivo* screen depends on genetic manipulation and selection, we reasoned that these two factors could be achieved by retroviral transduction in antigen-specific B cells and the selection of these B cells in GC responses. Here we show that retrovirally transduced antigen-specific B cells can be used to screen regulators for GC B cell differentiation *in vivo* and identify *Zdhhc2* as a novel positive regulator.

## Materials and Methods

### Mice

B1-8^hi^ (B6.129P2-PtrpcaIghtm1Mnz/J) mice were purchased from the Jackson laboratory. Wild-type C57BL/6 mice were purchased from Shanghai SLAC Laboratory Animal Company. All mice were maintained in a specific-pathogen-free animal facility at Shanghai Jiao Tong University School of Medicine (SJTUSM).

### Retroviral Constructs

The shRNA sequences were either designed by the Broad Institute GPP Web Portal or reported previously (50). The retroviral shRNA library was constructed by inserting the mix of shRNA double-strand fragments with 5′-BamHI and 3′-EcoRI sticky ends into the pSIREN-RetroQ_mCherry retroviral vector, in which the puromycin-resistant gene of pSIREN-RetroQ (Clontech) was replaced by the mCherry sequence from the mCherry-pBAD vector (Addgene).

For the study of *Bcl6*–shRNA and *Zdhhc2*-shRNA, *Bcl6*–shRNA (5′- GATCCGCTGTCAAAGAGAAGGCTTTATTCAAGAGATAAAGCCTTCTCTTTGACAGCTTTTTTGATATCG-3′) was inserted into the retroviral pSIREN-RetroQ_GFP vector, in which the puromycin-resistant gene of pSIREN-RetroQ was replaced by the green fluorescent protein (GFP) gene sequence from the PMKO.1-GFP vector (Addgene), *Zdhhc2*-shRNA-2 (5′-GATCCGTGACAGATGCCAACTTATAATTCAAGAGATTATAAGTTGGCATCTGTCACTTTTTTGATATCG-3′) and *Zdhhc2*-shRNA-4 (5′-GATCCGCTACTCCTGCGGGACTAAATTTTCAAGAGAAATTTAGTCCCGCAGGAGTAGCTTTTTTGATATCG-3′) were inserted into the retroviral pSIREN-RetroQ_mCherry vector, and a scramble shRNA (5′- GATCCGTGCGTTGCTAGTACCAACCTATTCAAGAGATAGGTTGGTACTAGCAACGCACTTTTTTGATATCG-3′) was inserted into the retroviral pSIREN-RetroQ_mCherry vector as controls.

### Retroviral Transduction and Analysis of Transduced B1-8^hi^ Cells

For *in vivo* screen, the retroviruses of shRNA library including 78 candidate genes were packaged in Phoenix cells; B1-8^hi^ splenic cells were stimulated with anti-CD180 (0.25 μg/ml, clone RP/14, BD Bioscience) for 24 h, then spin-infected at 2,000 *g* for 1.5 h with retroviruses in the presence of polybrene (8 μg/ml) (TR-1003-G, Millipore), and cultured overnight before transferring into eight wild-type C57BL/6 mice by tail vein injection (5~10 × 10^6^ cells per mouse). The recipients were immunized intraperitoneally with 100 μg of NP_49_-CGG (Biosearch Technologies, N-5055E) in Alum (Pierce, 77,161) per mouse the day after transfer. The GC B cells and the non-GC B cells were MACS-sorted [according to (51)] from splenic cells pooled from eight recipients at 10 days later. The total genomic DNA was extracted from sorted GC B cells and non-GC B cells, and each template was amplified five times in parallel. The shRNA fragments were amplified by nested PCR and subjected to next-generation sequencing. The primers used for the nested PCR are 5′-GAAGAGGGCCTATTTCCCATGATTC-3′ and 5′-ACTTCCATTTGTCACGTCCTGCAC-3′ for the 1st round of PCR, 5′-GGACTATCATATGCTTACCGTAACTTGA-3′ and 5′-TGGATGTGGAATGTGTGCGA-3′ for the 2nd round of PCR. The shRNA fragments were subjected to next-generation sequencing (Illumina Hiseq X Ten). Two independent screens were performed.

For the study of *Bcl6*–shRNA and *Zdhhc2*-shRNA, the retroviruses containing these gene-specific shRNAs and their scramble shRNA or empty vector controls were packaged in Phoenix cells. The B1-8^hi^ cells were cultured as described above and separately transduced with retroviruses containing these gene-specific shRNAs and scramble shRNAs tagged with mCherry reporter for *Zdhhc2*-shRNA or empty vectors tagged with GFP reporter for *Bcl6*–shRNA. The B1-8^hi^ cells transduced with the gene-specific or matched scramble shRNAs or empty vectors were mixed at a ratio of 1:1, with the B1-8^hi^ cells transduced with the empty vectors tagged with the alternative fluorescent reporter and adoptively transferred into wild-type C57BL/6 recipient mice (5~10 × 10^6^ cells per mouse), which were immunized intraperitoneally with 100 μg of NP_49_-CGG/Alum on the day after transfer, and analyzed by flow cytometry 10 days after immunization.

### NGS Data Processing and Analysis

To analyze the abundance of each shRNA fragment in the next-generation sequencing data, AWK (https://www.gnu.org/software/gawk/manual/gawk.html) and Python scripts were used to count the occurrence of each shRNA segment of the ~400 shRNA vectors in the five parallel data sets of each GC and non-GC B cell sample. The percentages of each shRNA fragment in the five parallel data sets of each sample were calculated, and the median value was used to represent its abundance in the sample. The shRNAs with median percentage values exceeding 0.025% (10 times less than the average abundance of 400 shRNA) in either non-GC or GC B cells were further analyzed to screen genes that specifically impact on GC B cells. The fold change was calculated as the GC/non-GC ratio for the abundance of each shRNA construct. The fold change values, expressed as binary logarithm (log2), and the reciprocals of their standard deviations were used to identify shRNA constructs with large and consistent (large reciprocal values) impacts on GC B cell differentiation.

### Flow Cytometry

The flow cytometry analysis was performed using the BD LSRFortessa™ X-20 analyzer (BD Biosciences). Mouse splenic cells were stained with anti-B220 (RA3-6B2, BioLegend), CD38 (90, eBioscience), CD95 (Jo2, BD), CD45.1 (A20, BioLegend), and CD45.2 (104, BioLegend) for the *in vivo* study of B1-8^hi^ cell differentiation or with anti-B220, CD95, GL7 (GL7, BioLegend), CD138 (281-2, BioLegend), biotin anti-mouse IgG1 (RMG1-1, BioLegend), and BV785 streptavidin (BioLegend) for the *in vitro* analysis of GC B cell and plasma cell differentiation in the co-culture system or with anti-B220, annexin V (BD), and 7AAD (BD) for the analysis of cell apoptosis. The transduced A20 cell lines (kindly provided by Dr. Jeffrey Ravetch, Rockefeller University) were stained with anti-mouse BCL6 (K112-91, BD) for the knockdown efficiency of *Bcl6*-specific shRNA.

### Genomic DNA Extraction

The sorted GC B cells were lysed in 10 mM Tris-HCl, pH 8.0, and 0.1 mM ethylenediaminetetraacetic acid (EDTA), proteinase K (B600452, Sangon Biotech), was added at a concentration of 0.5 mg/ml; the mixture was incubated at 50°C for 2.5 h and then at 95°C for 10 min. The sorted non-GC B cells were lysed in genomic DNA buffer without sodium dodecyl sulfate (SDS; 100 mM NaCl, 10 mM Tris-HCl, pH 8.0, and 1 mM EDTA), mixed with an equal volume of genomic DNA buffer with 1% SDS and 0.2 mg/ml proteinase K, and incubated for 4 h at 37°C on a shaking heating block (Allsheng). Isopropanol was added to precipitate genomic DNA, which was further washed with 70% ethanol and resolved in ddH_2_O. Both GC B cell lysates and purified non-GC B cell genomic DNA were used as templates to amplify the shRNA fragments.

### Quantitative PCR

RNA was prepared with TRIzol (Invitrogen) from the GC B cells and the non-GC B cells of Peyer's patches by fluorescence-activated cell sorting. Quantitative real-time PCR was performed with One-Step SYBR PrimeScript RT-PCR Kit II (Perfect Real Time) (RR086A, Takara) and a 7,500 Fast Real-Time PCR System (Applied Biosystems). Gene expression was normalized to that of β-actin, and data were presented as a fold difference of the normalized values relative to that of the control samples by ‘2^−ΔΔCT^’ (change in cycling threshold). The following primer sets were used:

β*-actin* forward: 5′-CGCCACCAGTTCGCCATGGA-3′,
β*-actin* reverse: 5′-TACAGCCCGGGGAGCATCGT-3′,
*Zdhhc2* forward: 5′-TGGTCTGCCTGATACTCAAGCCAAG-3′,
*Zdhhc2* reverse: 5′- CTGAAACCCAAGCTGAATCCGTTC-3′.

### *In vitro* Co-culture of B Cells

Wild-type (WT) C57BL/6 splenic single cells were prepared and cultured in RPMI-1640 medium supplemented with 10% FBS (Gibco), 2 mM L-glutamine (Gibco), 1% Pen/Strep (100 units/ml penicillin, 100 μg/ml streptomycin, Hyclone), 10 mM HEPES (pH 7.2–7.5, Gibco), 1 mM sodium pyruvate (Gibco), 1% non-essential amino acids (Gibco), and 50 μM 2-mercaptoethanol. Splenocytes (1 × 10^5^ cells per well) were co-cultured overnight with NB-21.2D9 feeder cells (irradiated with 8 Gy X-ray, 1.5 × 10^4^ cells per well) in a 24-well plate (Corning), together with 2 ng/ml mIL-4 (Sino Biological). These cells were then spin-infected with retroviruses containing *Zdhhc2*- or scramble shRNAs at 2,000 *g* for 1.5 h and further cultured at the same condition. B cell differentiation and apoptosis were analyzed by flow cytometry at 48 h after transduction. To analyze cell proliferation, the transduced B cells were labeled with carboxyfluorescein succinimidyl ester (CFSE) (Invitrogen) at 48 h after transduction and further co-cultured with irradiated NB-21.2D9 feeder cells in the presence of 2 ng/ml mIL-4 for 2 days before the flow cytometry analysis.

### Statistical Analysis

All statistical tests were performed using the GraphPad Prism 6.0 software. The specific statistical test for each experiment is described in the figure legends. Unpaired *t*-test with a two-tailed 95% confidence interval was employed for the comparison of two groups. One-way ANOVA with Dunnett's test or two-way ANOVA with Sidak's test was applied to multiple comparisons. A *P* value <0.05 was considered as statistically significant.

## Results

### Establishment of an *in vivo* shRNA Screening System Based on B1-8^hi^ Cells

To establish an *in vivo* screening system for B cell-intrinsic factors that regulate GC B cell differentiation, we hypothesized to exploit the clonal expansion feature of antigen-specific GC B cells and a retroviral shRNA system that has been previously described (52). To validate the system, we first confirmed that B1-8^hi^ cells, an established NP-specific B cell response model (21), can differentiate into GC B cells *in vivo*, both before and after *in vitro* retroviral transduction and adoptive cell transfer. As shown in Supplemental Figure 1, fresh B1-8^hi^ cells expanded from 0.13% in the non-GC B cell compartment to ~48% in the GC B cell compartment when adoptively transferred into WT recipient mice and challenged with NP-CGG/Alum, suggesting that fresh B1-8^hi^ cells can efficiently differentiate into GC B cells. At the same time, retrovirally transduced B1-8^hi^ cells could also differentiate into GC B cells, although at a lower efficiency, as shown by an expansion from 0.79% in the non-GC B cell compartment to 2.96% in the GC B cell compartment.

**Figure 1:**
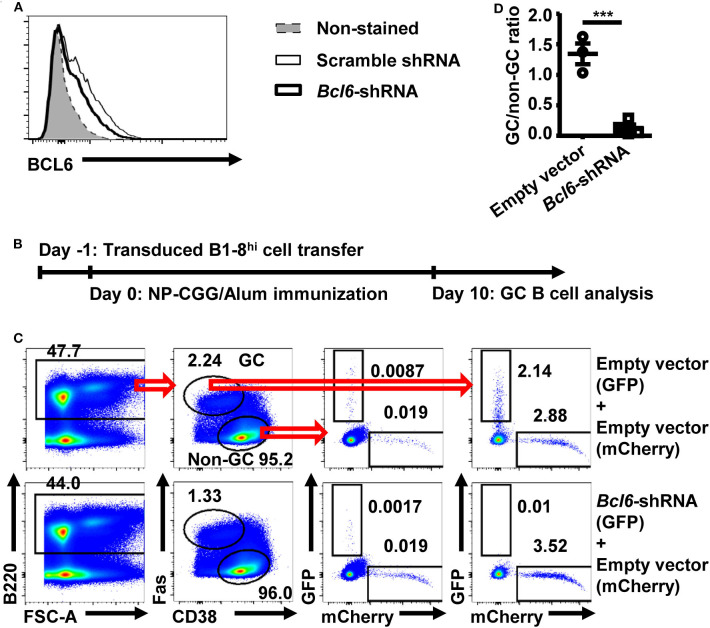
Retrovirally transduced B1-8^hi^ cells can be used to study the shRNA function. **(A)** Representative flow cytometry profile showing the intranuclear BCL6 levels in untransduced A20 cells or transduced A20 cells with scramble shRNA or *Bcl6*–shRNA retroviral vectors. Murine B cell lymphoma A20 cells were either untransduced or transduced with the indicated retroviral vectors and analyzed for intranuclear BCL6 levels by flow cytometry 3 days later. **(B)** B1-8^hi^ cell transfer model for evaluating germinal center (GC) B cell differentiation. Fresh or retrovirally transduced B1-8^hi^ cells were adoptively transferred to wild-type (WT) recipients on day−1, immunized with NP-CGG/Alum on day 0, and analyzed for GC B cell differentiation on day 10. **(C,D)** Representative flow cytometry profiles **(C)** showing the percentages of empty vector and *Bcl6*–shRNA transduced B1-8^hi^ cells (expressing a GFP reporter) as well as the co-transferred empty vector transduced B1-8^hi^ cells (expressing a mCherry reporter) in non-GC B cells and GC B cells, and representative graph **(D)** showing the GC/non-GC ratios of percentages of transduced B1-8^hi^ cells (expressing GFP) normalized to empty vector transduced cells (expressing mCherry). The empty vector or *Bcl6*–shRNA transduced B1-8^hi^ cells (expressing GFP) were mixed 1:1 with empty vector transduced B1-8^hi^ cells (expressing mCherry) and adoptively transferred into WT recipient mice and evaluated for their percentages among non-GC B cells and GC B cells **(C)** 10 days after NP-CGG/Alum immunization. The ratio of the percentage of empty vector or *Bcl6*–shRNA transduced cells (expressing GFP) in GC B cells to that in the matched non-GC B cells was calculated and normalized to the ratio of empty vector transduced cells (expressing mCherry), expressed as GC/non-GC ratio **(D)**. Each symbol in **(D)** represents data from an individual mouse. The bars represent mean ± SEM; ***p ≤ 0.001, unpaired two-tailed *t*-test. A representative of two independent experiments is shown.

To validate an *in vivo* screening system based on retroviral shRNA knockdown, a shRNA vector targeting *Bcl6* was tested. The *Bcl6*–shRNA was confirmed to efficiently knockdown BCL6 expression (Figure 1A). As illustrated in Figure 1B, B1-8^hi^ cells transduced with the *Bcl6*–shRNA vector containing a GFP reporter were mixed with B1-8^hi^ cells transduced with empty vector containing a mCherry reporter, adoptively transferred into WT recipient mice, and evaluated for their expansion in the GC B cell compartment 10 days after NP-CGG/Alum immunization. As shown in Figure 1C, while the empty vector-transduced cells were present in both non-GC and GC B cell compartments, the *Bcl6*–shRNA transduced cells were almost completely absent from the GC B cell compartment, but not in the non-GC B cell compartment, which led to a significantly reduced GC/non-GC ratio for *Bcl6*–shRNA transduced cells (Figure 1D). While these results are consistent with the previous report that *Bcl6* is essential for GC B cell differentiation (28), they also provide the basis for the use of retroviral shRNA transduced B1-8^hi^ B cells for *in vivo* screen.

**Figure 2:**
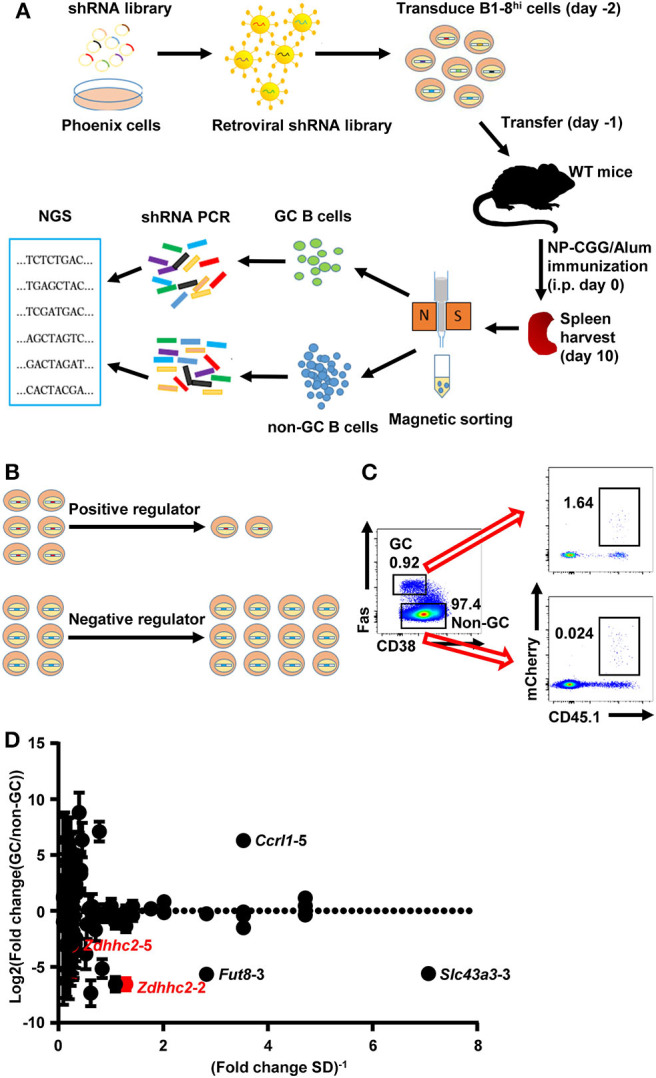
Overview of the shRNA *in vivo* screening system for regulators of germinal center (GC) B cell differentiation. **(A)** A schematic view of the *in vivo* screening system for regulators of GC B cell differentiation. The shRNA retroviral library was produced in Phoenix cells and used to transduce B1-8^hi^ cells on day−2, which were adoptively transferred into eight wild-type (WT) recipient mice on day−1. These mice were immunized with NP-CGG/Alum on day 0, sacrificed on day 10, and subjected to splenic GC and non-GC B cell isolation by magnetic sorting. The shRNA sequences were amplified by PCR and determined by next-generation sequencing to evaluate the abundance of each shRNA construct. **(B)** Illustration of the screening strategy: shRNA constructs that target the positive regulators of GC B cell differentiation are less abundant and *vice versa* for negative regulators. **(C)** Representative flow cytometry profiles showing the percentages of retroviral shRNA library transduced cells (expressing mCherry) in GC and non-GC B cells in mice treated as described in **(A)**. **(D)** Plot of the binary logarithm of shRNA's fold change values (calculated as the ratio of each shRNA's abundance in GC B cells to that in the corresponding non-GC B cells) against the reciprocals of their standard deviations [(fold change SD)^−1^]. Two independent screens were performed.

To test whether retroviral shRNA transduced B1-8^hi^ cells can be used for *in vivo* screen, a small shRNA library of ~400 shRNA retroviral constructs targeting 78 candidate genes (five shRNA constructs for each gene, Supplemental Table 1) was constructed and confirmed by next-generation sequencing, with ~95% constructs comprising more than 0.025% of all sequences (Supplemental Figure 2). These genes are differentially expressed in GC and follicular B cells based on the Immunological Genome Project database (http://rstats.immgen.org/PopulationComparison/index.html) and based on the scarcity of information regarding their impacts on GC B cell differentiation. As illustrated in Figure 2A, B1-8^hi^ B cells transduced with the shRNA library were transferred into WT recipient mice and challenged with NP-CGG/Alum immunization. The transduction efficiency of B1-8^hi^ cells with the library is ~ 24% as analyzed 24 hours or 43~72% as analyzed 48 hours after transduction (Supplemental Figure 3). The GC B cells and the non-GC B cells were sorted 10 days later and analyzed for the abundance of these shRNA constructs in these cells. We reasoned that shRNA constructs that target the critical positive regulators of GC B cell differentiation will be less abundant and *vice versa* for the critical negative regulators (Figure 2B).

As shown in Figure 2C, a small percentage of GC B cells expressing fluorescent reporter genes (mCherry) was observed, suggesting that the retroviral constructs had successfully transduced these cells. The total number of retrovirally transduced B cells is ~10 times more than that of the shRNA constructs, providing the basis for a further screen. The shRNA fragments were amplified from these cells and subjected to next-generation sequencing. To minimize the variation, each sample was amplified and sequenced five times in parallel, and the median percentage values of each shRNA fragment were used to represent its abundance. To screen for genes that specifically impact on GC B cell differentiation, we focused on shRNA constructs that consistently recovered in either the non-GC or the GC B cell compartment. Based on two independent experiments, 85 shRNA constructs (targeting 52 out of 78 candidate genes) fall into this category (Supplemental Table 2). The fold change, defined as the abundance of each shRNA construct in GC B cells relative to the corresponding non-GC B cells, was plotted against the reciprocal of its standard deviation to help in the identification of shRNA constructs with large and consistent impacts on GC B cell differentiation. As shown in Figure 2D, B1-8^hi^ cells transduced with several shRNAs (*Zdhhc2*-2, *Fut8*-3, *Slc43a3*-3, *etc*.) had a consistently reduced differentiation to GC B cells relative to non-GC B cells, suggesting that they target potential positive regulators. In contrast, B1-8^hi^ cells transduced with some shRNA constructs (*Ccrl1*-5, *etc*.) that transduced cells were observed to be relatively more abundant in GC B cells than in non-GC B cells, suggesting their potential roles in negatively regulating GC B cell differentiation. Other shRNAs for these genes (*Zdhhc2, Fut8, Slc43a3*, and *Ccrl1*) are either lost in both screens (*Zdhhc2*-1, *Fut8*-2,5, *Slc43a3*-1,5, and *Ccrl1*-1,2,3,4) or only recovered in one experiment (*Zdhhc2*-3,4, *Fut8*-1,4, and *Slc43a3*-2,4), except for *Zdhhc2*-5, which displayed variable levels of inhibition of GC B cell differentiation (Figure 2D).

### *Zdhhc2* shRNA Is Verified to Inhibit GC B Cell Differentiation

**Figure 3:**
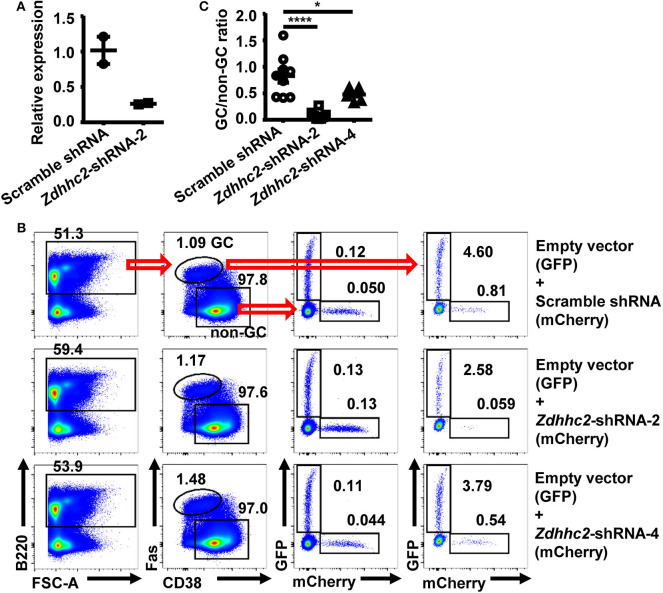
Reduced *in vivo* differentiation of *Zdhhc2*-shRNA transduced B cells into germinal center (GC) B cells. **(A)** Graph showing the relative mRNA levels in scramble shRNA and *Zdhhc2*-shRNA transduced B cells. Each symbol represents an independent culture. **(B,C)** Representative flow cytometry profiles **(B)** showing the precentages of scramble shRNA or *Zdhhc2*-shRNA transduced B1-8^hi^ cells (expressing mCherry) as well as the co-transferred empty vector transduced B1-8^hi^ cells (expressing GFP) in non-GC B cells and GC B cells, and representative graph **(C)** showing the GC/non-GC ratio of percentages of scramble shRNA or *Zdhhc2*-shRNA transduced B1-8^hi^ cells (expressing mCherry) normalized to empty vector transduced cells (expressing GFP), analyzed as described in Figures 1C,D. Each symbol in **(C)** represents data from an individual mouse. The bars represent mean ± SEM; *p ≤ 0.05, ****p ≤ 0.0001, one-way ANOVA with Dunnett's multiple-comparisons test. A representative of two independent experiments is shown.

To verify the *in vivo* screening results, *Zdhhc2* shRNA was further studied, given its strong activity as observed in the screen. The knockdown effect of *Zdhhc2*-shRNA-2 was confirmed in *in vitro* differentiated B cells with GC B cell phenotype (53), where *Zdhhc2*-shRNA-2 transduced cells displayed significantly reduced *Zdhhc2* mRNA levels (Figure 3A), consistent with the results of previous studies on this shRNA (50). *Zdhhc2*-shRNA-2 transduced B1-8^hi^ cells (mCherry^+^) were cotransferred with empty vector transduced B1-8^hi^ cells (GFP^+^) into WT recipient mice and evaluated for their expansion in the GC B cell compartment 10 days after NP-CGG/Alum immunization (Figure 1B). While comparable levels of *Zdhhc2*-shRNA-2 and empty vector transduced cells were observed in non-GC B cells, the *Zdhhc2*-shRNA-2 transduced B1-8^hi^ cells were essentially absent in GC B cells (Figure 3B) and had significantly reduced GC/non-GC ratios (Figure 3C). To further validate that *Zdhhc2* is a positive regulator, *Zdhhc2*-shRNA-4, another *Zdhhc2*-shRNA described previously (50), was also tested. As shown in Figures 3B,C, *Zdhhc2*-shRNA-4 could also significantly reduce the differentiation of B1-8^hi^ cells into GC B cells. These data suggest that *Zdhhc2* is a positive regulator of GC B cell differentiation.

**Figure 4:**
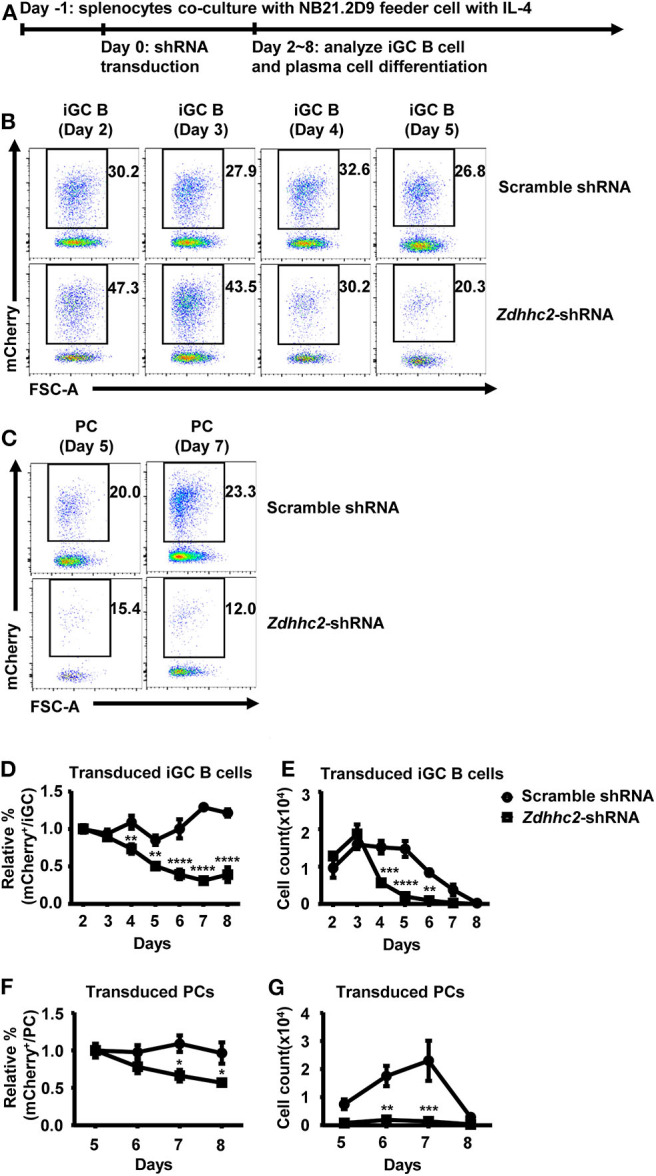
Reduced *in vitro* differentiation of *Zdhhc2*-shRNA transduced B cells into germinal center (GC) B cells and plasma cells. **(A)** The *in vitro* model for analyzing iGC B cell and plasma cell differentiation. The wild-type (WT) splenic cells were co-cultured with irradiated NB-21.2D9 feeder cells (expressing CD40L, BAFF, and IL21) in the presence of 2 ng/ml of IL-4 on day−1, transduced with scramble shRNA or *Zdhhc2*-shRNA-2 retroviral vectors (expressing mCherry) on day 0, and analyzed for iGC B cell and plasma cell differentiation from day 2 to day 8. **(B,C)** Representative flow cytometry profiles showing the percentages of scramble shRNA or *Zdhhc2*-shRNA transduced cells (mCherry^+^) among iGC B cells (B220^+^Fas^+^GL7^+^) as cultured in **(A)** on days 2–5 **(B)** and plasma cells (B220^+^CD138^+^IgG1^+^ or PC) on day 5 and day 7 **(C)**. **(D–G)** Representative graphs showing the relative percentage **(D,F)** or the absolute number **(E,G)** of scramble shRNA or *Zdhhc2*-shRNA transduced cells among iGC B cells (normalized to the values on day 2) **(D,E)** or plasma cells (normalized to the values on day 5) **(F,G)** at the indicated time points (days 2–8 for iGC B cells, days 5–8 for PC) in the cultures described as in **(A)**. The bars represent mean ± SEM. *p ≤ 0.05, **p ≤ 0.01, ***p ≤ 0.001, ****p ≤ 0.0001, two-way ANOVA with Sidak's multiple-comparisons test. A representative of two independent experiments is shown.

To further investigate the function of *Zdhhc2* in GC B cell differentiation, a culture system in which mouse naïve B cells undergo extensive proliferation and differentiation into GC-phenotype B cells and plasma cells was exploited (53). In this system, the naïve B cells were co-cultured with NB21.2D9 feeder cell lines that express CD40L, BAFF, and IL-21. At 24 h after co-culture, the splenic B cells were transduced with *Zdhhc2*-shRNA or scramble shRNA retroviral vectors and analyzed for their differentiation (Figure 4A). Consistent with the previous report, the splenic B cells can differentiate into GC phenotype B cells (B220^+^Fas^+^GL7^+^, referred to as “iGC”), which are most abundant on day 4, and then to plasma cells (B220^+^CD138^+^IgG1^+^, referred to as “PC”) (Supplemental Figure 4). Importantly, the percentage and the number of *Zdhhc2*-shRNA-2 transduced iGC B cells were significantly reduced as compared to those of scramble shRNA transduced iGC B cells from as early as day 4 (Figures 4B,D,E). The reduction peaked at days 6 and 7 and is not due to the retroviral transduction process as the percentages of scramble shRNA transduced iGC B cells and plasma cells are almost maintained constant (Figures 4D,F). A quantification of the percentage and the number of plasma cells also supports that *Zdhhc2*-shRNA-2 transduced B cells have reduced differentiation into plasma cells (Figures 4C,F,G).

### *Zdhhc2* Is Highly Expressed in GC B Cells

**Figure 5:**
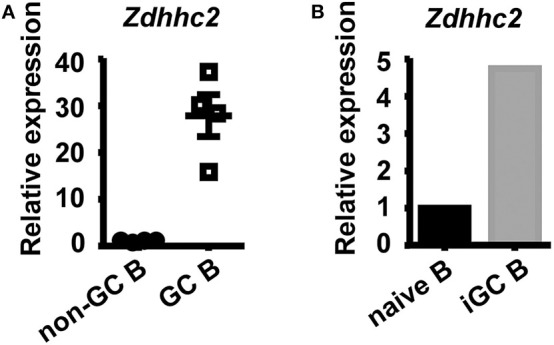
High expression levels of *Zdhhc2* in germinal center (GC) B cells. **(A,B)** Graphs showing the relative *Zdhhc2* mRNA levels, quantified by qRT-PCR, in non-GC B and GC B cells sorted by fluorescence-activated cell sorting (FACS) from Peyer's patches of wild-type mice **(A)**, FACS-sorted splenic naïve B cells, and induced GC B cells (iGC B) cultured as in Figure 4A. Each symbol in **(A)** represents the sample from an individual mouse. *N* = 1 in **(B)**.

To further explore the role of *Zdhhc2* in GC B cell differentiation, its expression was analyzed. As shown in Figure 5A, the mRNA levels of *Zdhhc2* in GC B cells, quantified by qPCR, are about 20-fold higher than those in follicular B cells. *Zdhhc2* expression is also induced in *in vitro* differentiated iGC B cells (Figure 5B). These results are consistent with previously published microarray and RNA-seq data (Supplemental Figure 5A) and data from the Immunological Genome Project (Supplemental Figures 5B,C) (54, 55).

### *Zdhhc2* Promotes B Cell Proliferation and Survival

**Figure 6:**
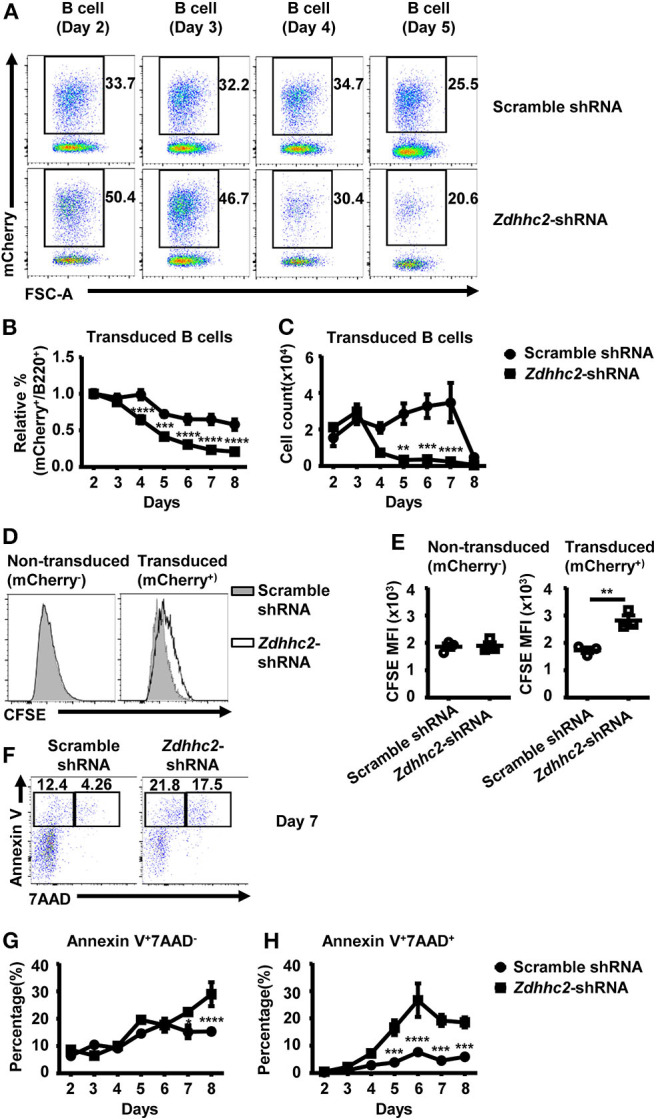
The reduced proliferation and the increased cell death in *Zdhhc2*-shRNA transduced B cells. **(A)** Representative flow cytometry profiles showing the percentages of scramble shRNA or *Zdhhc2*-shRNA transduced cells (mCherry^+^) among B cells (B220^+^) cultured as in Figure 4 on days 2–5. **(B,C)** Representative graphs showing the relative percentage (normalized to the values on day 2) **(B)** or the absolute number **(C)** of scramble shRNA or *Zdhhc2*-shRNA transduced cells among B cells at the indicated time points in the culture described as in Figure 4. **(D,E)** Representative flow cytometry profiles showing the carboxyfluorescein succinimidyl ester (CFSE) levels **(D)** and graphs showing the CFSE MFI levels **(E)** in scramble shRNA or *Zdhhc2*-shRNA transduced B cells (mCherry^+^) and non-transduced B cells (mCherry^−^). The splenic cells were cultured and transduced as described in Figure 4A on day −1 and day 0, then labeled with CFSE on day 2 and further co-cultured with irradiated NB-21.2D9 feeder cells, and analyzed for CFSE levels on day 4. **(F,G,H)** Representative flow cytometry profiles **(F)** and graphs showing the percentages of apoptotic cells (Annexin-V^+^7AAD^−^) **(G)** and dead cells (Annexin-V^+^7AAD^+^) **(H)** in scramble shRNA or *Zdhhc2*-shRNA transduced B cells (mCherry^+^). Each symbol represents an independent culture. The bars represent mean ± SEM. *p ≤ 0.05, **p ≤ 0.01, ***p ≤ 0.001, ****p ≤ 0.0001, two-way ANOVA with Sidak's multiple-comparisons test **(B,C,G,H)** or unpaired two-tailed *t*-test **(E)**. The data are representative of two independent experiments.

To further investigate how *Zdhhc2* knockdown impacts on GC B cell differentiation, we analyzed its impact on B cell proliferation and apoptosis. We noticed that the percentage and the number of *Zdhhc2*-shRNA transduced B-lineage cells were lower than those of scramble shRNA transduced cells in the *in vitro* culture system (Figures 6A–C), suggesting that *Zdhhc2*-shRNA may have an impact on B cell proliferation and survival. To investigate this possibility, *Zdhhc2*-shRNA transduced iGC B cells were labeled with CFSE and analyzed 2 days later. As shown in Figures 6D,E, *Zdhhc2*-shRNA transduced cells have significantly higher CFSE levels as compared to scramble shRNA transduced cells. In contrast, the co-cultured non-transduced cells have comparable CFSE levels (Figures 6D,E). These results suggest that *Zdhhc2*-shRNA transduction inhibits B cell proliferation in response to CD40L/BAFF/IL-21 stimulation. At the same time, increased percentages of apoptotic cells (annexin V^+^7AAD^−^), which was only observed from day 7, and dead cells (annexin V^+^7AAD^+^), from as early as day 5, were also observed among *Zdhhc2*-shRNA transduced cells as compared to the scramble shRNA transduced cells (Figures 6F–H). Collectively, these results suggest that *Zdhhc2* can regulate GC B cell differentiation by regulating both B cell proliferation and survival.

## Discussion

Here we show the proof of concept of a functional *in vivo* screen for genes that regulate GC B cell differentiation using retroviral shRNA vector transduced antigen-specific B cells. Despite that this approach only allows the screen of relatively small library given that only a small fraction of retrovirally transduced cells is present in the GC B cell compartment (~4,000 retrovirally transduced B1-8^hi^ cells were recovered in each screen, which is ~10 times more than that of the shRNA constructs). Because of this limitation, loss of some shRNAs in the screen is expected. At the same time, some shRNA constructs may be lost due to their biological activity, which could not be distinguished at this point. Regardless, some novel strong regulators of GC B cell differentiation can be identified. We noticed that the knockdown of *Bcl6* results in a more substantial reduction in GC B cells as compared to the reduction observed in heterozygous *Bcl6* mice (*vs*. WT mice), where the percentage of GC B cells is reduced to ~50% (56). We reasoned that in our system the *Bcl6*–shRNA transduced B1-8^hi^ cells need to compete with *Bcl6*-sufficient B1-8^hi^ cells in the same mice, where the selection disadvantage of *Bcl6*–shRNA transduced B1-8^hi^ cells might become more evident under such competition pressure, a phenomenon that we have previously observed (57). Therefore, one advantage of our system is to provide the sensitivity of competition models. This system provides a new way to discover genes that regulate GC B cell differentiation and perhaps post-GC B cell differentiation, such as memory and plasma cell differentiation.

The *in vivo* screening system may be further optimized in the future for the higher-throughput screen. It is noted that while the retroviral transduction efficiency can reach ~50%, as assessed by flow cytometry at 2 days after transduction, the efficiency of these retrovirally transduced B1-8^hi^ cells is reduced as compared to that of fresh B1-8^hi^ cells without the retroviral transduction procedure, suggesting that the stimulation during the retroviral transduction procedure is detrimental, to some extent, for GC B cell differentiation. It is possible that moving the retroviral transduction to earlier B cell development stages may help to optimize the system, which is the subject of a future study.

The identification of *Zdhhc2* in our screen suggests that *Zdhhc2* is a strong regulator of GC B cell differentiation. As far as we know, no study of *Zdhhc2* in B cells has been described. As a member of DHHC-palmitoyl transferases, *Zdhhc2* has been mainly studied in the field of cancer and neuroscience (58, 59). In humans, aberrant ZDHHC2 expression or translocation has been described in several cancers, including acute myeloid leukemia and hepatocellular carcinoma (59). Palmitoylation mediated by DHHC-palmitoyl transferases has been proposed to regulate oncogenic RAS signaling and tumor suppressor localization to the plasma membrane (59). ZDHHC2 has also been implicated in mediating the palmitoylation of PSD-95 and AKAP79/150 required for neuronal activity (60, 61). The reported substrates of ZDHHC2 also includes CD9 and CD151 in HEK293 cells (62), Lck in T cells (63), and CKAP4 in hepatocellular carcinoma and interstitial cystitis (64–66).

The function of *Zdhhc2* in B cell is, however, poorly understood. Palmitoylation of CD81 has been found to be necessary for the raft-stabilizing function of the CD19/CD21/CD81 complex to facilitate BCR signaling (67). The B lymphocyte-specific immune regulators CD20 and CD23 have been identified as novel palmitoylated proteins in human B cells (68). Since the reversible cycles of palmitoylation and depalmitoylation have been proposed to regulate the precise membrane localization of proteins, membrane association, protein stability and traffic, and cell signaling processes (58, 69–75), we speculate that *Zdhhc2* may regulate GC B cell differentiation through modulating the palmitoylation of unknown targets associated with cell proliferation and survival in B cells. The underlying molecular mechanism has remained to be revealed.

## Data Availability Statement

The raw data supporting the conclusions of this article will be made available by the authors, without undue reservation, to any qualified researcher.

## Ethics Statement

The animal study was reviewed and approved by SJTUSM Institutional Animal Care and Use Committee.

## Author Contributions

FL and RZ designed the study and analyzed the results and wrote the paper. RZ performed the experiments. HZ, YZ, and CH provided technical supports. DL provided next-generation sequencing data processing supports. All authors reviewed and approved the manuscript.

## Conflict of Interest

DL was employed by the company Boston Consulting Group. The remaining authors declare that the research was conducted in the absence of any commercial or financial relationships that could be construed as a potential conflict of interest.
