# Supplementary material for: In vivo Screen Identifies Zdhhc2 as a Critical Regulator of Germinal Center B Cell Differentiation
Source: Front Immunol. 2020 Jun 10;11:1025. doi: 10.3389/fimmu.2020.01025 (PMC7297983; doi:10.3389/fimmu.2020.01025)
Supplement: Supplementary file 1 [file Data_Sheet_1.docx]

**
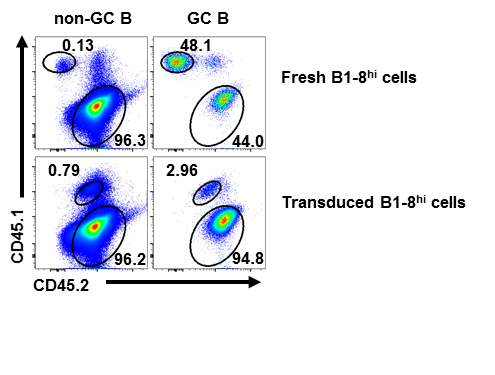
Supplementary Figure 1.** Differentiation of fresh and transduced B1-8^hi^ into GC B cells *in vivo*. Representative flow cytometry profiles showing the percentages of fresh (CD45.1^+/+^) or transduced (CD45.1^+^CD45.2^+^) B1-8^hi^ cells in non-GC or GC B cells. Indicated B1-8^hi^ cells were adoptively transferred to WT mice (CD45.2^+/+^) on day -1, which were immunized with NP-CGG/Alum on day 0 and analyzed on day 10 for the percentages of B1-8^hi^ cells in non-GC B and GC B cells.

**
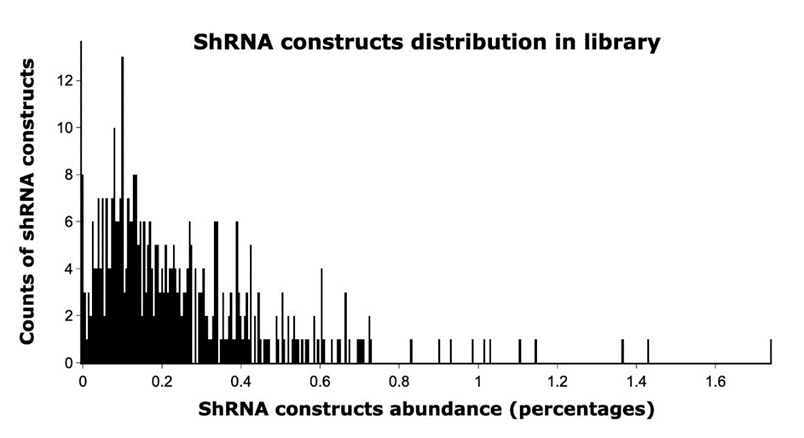
**

**Supplementary Figure 2.** The histogram plot showing the distribution of the abundance of ~400 shRNA constructs in the shRNA library confirmed by next-generation sequencing. The abundance (percentages) range of shRNA constructs is divided into 400 bins with each bin representing 0.004% (X axis), and the number of shRNA constructs falling into each bin are counted (Y axis).

**
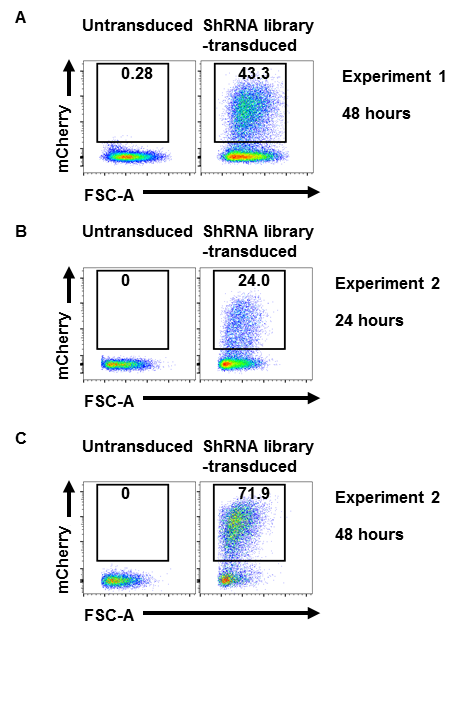
Supplementary Figure 3**. The flow cytometry profiles showing the percentages of shRNA library transduced B1-8^hi^ cells among B cells as analyzed 48h after transduction **(A)** in Experiment 1 and 24h **(B)** or 48h after transduction **(C)** in Experiment 2 of two independent screens.

**
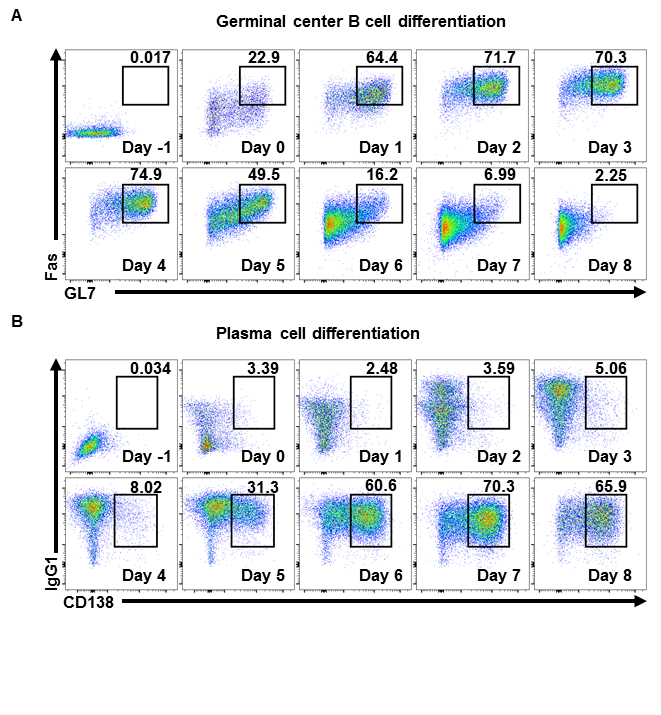
Supplementary Figure 4.** *In vitro* differentiation of splenic B cells into GC B cells and plasma cells. **(A)**, **(B)**. Representative flow cytometry profiles showing the percentages of iGC B cells (Fas^+^GL7^+^) **(A)** and plasma cells (CD138^+^IgG1^+^) **(B)** in B lineage cells (B220^+^ or B220^low^) cultured as described in Figure 4.

**
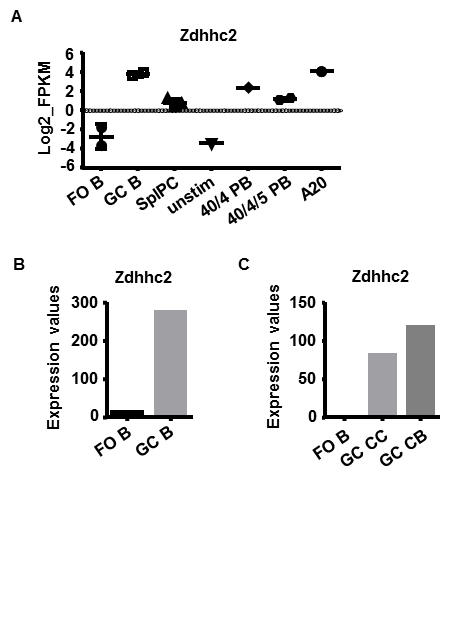
Supplementary Figure 5.** *Zdhhc2* mRNA expression levels documented in reference paper and databases. **(A)**. *Zdhhc2* mRNA expression levels in different B cell subsets reported in a published dataset (GEO accession number: GSE60927) (54). Follicular B cells (FO B), GC B cells (GC B), splenic plasma cells (SplPC), Small resting B cells from Blimp1-GFP mice (unstim), Day 4 CD40L+IL4 stimulated cells, Syndecan1^+^ (40/4 PB), Day 5 CD40L+IL4+IL5 stimulated cells, Syndecan1^+^ (40/4/5 PB), GC-like B lymphoma cell line (A20). **(B)** and **(C)**. *Zdhhc2* mRNA expression levels in follicular B cells (FO B), germinal center B cells (GC B), centrocytes (CC), and centroblasts (CB), micro-array **(B)** and RNA-seq **(C)** data from ImmGen Consortium (55).

References:

54. Shi W, Liao Y, Willis SN, Taubenheim N, Inouye M, Tarlinton DM, et al. Transcriptional profiling of mouse B cell terminal differentiation defines a signature for antibody-secreting plasma cells. *Nat Immunol* (2015) 16:663-73.doi: 10.1038/ni.3154.

55. Heng TS, Painter MW, Immunological Genome Project C. The Immunological Genome Project: networks of gene expression in immune cells. *Nat Immunol* (2008) 9:1091-4.doi: 10.1038/ni1008-1091.

**Supplementary Table 1.** List of shRNAs

| ShRNA | Target sequence (5’→ 3’) |
| --- | --- |
| Scramble shRNA | GTGCGTTGCTAGTACCAACCTA |
| *Bcl6* | GCTGTCAAAGAGAAGGCTTTA |
| *Cd86* | GCACAGAGAAACTTGATAGTGT |
| *Cd40* | GCACACTCAACCTTTATATTTAC |
| *Tnf* | GCACAACCAACTAGTGGTGCTT |
| *Inpp5d* | GGAAGTCATCAGGACTCTGCA |
| *Fcgr2b* | AGGGATGCTGTAGATATTAAA |
| *Aicda* | GCGAGATGCATTTCGTATGTT |
| *Efhd2* | GCGTTTGCCTCAGCGGATA |
| *Efnb1* | GCACTGTGCTTGATCCCAAT |
| *Arhgap11a*-1 | GCAGCAATCTTGCAGTAATAT |
| *Arhgap11a*-2 | GCTCGTCATCAGTGTAAATAA |
| *Arhgap11a*-3 | AGCAATCTTGCAGTAATATTT |
| *Arhgap11a*-4 | CCAAAGTCAGTCGTAAAGAAA |
| *Arhgap11a*-5 | CCTTTGGATGATCTCACGAAT |
| *Pask*-1 | GTGAGGGCGAGTACGACTATA |
| *Pask*-2 | GCTGCCAGATGGCACAATTTA |
| *Pask*-3 | AGCAGTGTGGACACGTGTAAT |
| *Pask*-4 | CCAGTCCTTAAAGAACATTTA |
| *Pask*-5 | CCTGAGGTTCTCATTGGAAAT |
| *Rgs9*-1 | CCCAAGAAAGTAGCCAACTTT |
| *Rgs9*-2 | CCCAAGTGCATTAGGATAATA |
| *Rgs9*-3 | CCTTCCAAGCAATCCTTGGAT |
| *Rgs9*-4 | CCGATTTCAGACGCCATATTT |
| *Rgs9*-5 | CCTGGAATGAACAATGTGTTA |
| *Smco4*-1 | TGGTCTTTGACTTGATCAATA |
| *Smco4*-2 | GAGCACGTCCATTCTGCTTTC |
| *Smco4*-3 | TGCTCCTCATTGTCGTGTTTG |
| *Smco4*-4 | CAACTCAAAGGGAAGCCAAAG |
| *Smco4*-5 | GTGCTCCTCATTGTCGTGTTT |
| *Akr1e1*-1 | ATCCTTTCCACATAGAGTATT |
| *Akr1e1*-2 | GAATATCCAGGTATTTGATTT |
| *Akr1e1*-3 | GCACTTCGATTGTGCTTACTT |
| *Akr1e1*-4 | CCATGAGATGGTTTCAGTAAC |
| *Akr1e1*-5 | CCAAAGGAACTTAATAGTGAT |
| *Fut8*-1 | GCTGGTGTGTAACATCAATAA |
| *Fut8*-2 | TGGTCATTTGGTTCGAGATAA |
| *Fut8*-3 | GCCCATACACAGTACAATAAT |
| *Fut8*-4 | CGCAGAATGCAAGTGGATAAA |
| *Fut8*-5 | CGAGATAATGACCACCCTGAT |
| *Anubl1*-1 | TATGCGTGGTGGACCGATAAG |
| *Anubl1*-2 | ATACAGTTGCATACAACATAT |
| *Anubl1*-3 | TAAGAGTCCTCCCTCATATTG |
| *Anubl1*-4 | TTCCTCAGTTGACCCATATAG |
| *Anubl1*-5 | GATGACAATTGGGAGATTAAC |
| *Dck*-1 | GCAGAGAAACCTGTATTATTT |
| *Dck*-2 | GCCTTGAATTGGATGGAATAA |
| *Dck*-3 | GCGGTGGAAATGTTCTTCAAA |
| *Dck*-4 | CTGGATGTTAATGAAGACTTT |
| *Dck*-5 | CTCAAGAGGAATTTGAGGAAT |
| *Rgs10*-1 | CACACCCTCTGATGTTCCAAA |
| *Rgs10*-2 | CAGCGAAGAGAATGTCTTGTT |
| *Rgs10*-3 | CCTCCAGAATTTACAACACAT |
| *Rgs10*-4 | CTGTCATTTCTTTGTTGTGTT |
| *Rgs10*-5 | CTTCTTGAAGTCTGACTTGTT |
| *Kcnn4*-1 | ATGCTAGGAAGCTTCAGTTAA |
| *Kcnn4*-2 | CATGGCCACCACTACACATAT |
| *Kcnn4*-3 | TTCATGATGGACATCCATTAT |
| *Kcnn4*-4 | AGTTCAACAAGGCGGAGAAAC |
| *Kcnn4*-5 | CTGCTCCTGGTTAAGTGTTTG |
| *Slc43a3*-1 | CCTGGCATCCTTCATGAATAA |
| *Slc43a3*-2 | CCTCGTCATTAAGCTGCTTTA |
| *Slc43a3*-3 | CCTTAAACAGAAGTATCAGAA |
| *Slc43a3*-4 | CCTACAGATTGGGAACCTCTT |
| *Slc43a3*-5 | TCTAGCCTCGATGATGCCTTT |
| *Slc22a15*-1 | GCGACATCATATCCGCAAATT |
| *Slc22a15*-2 | GCTGCCTTTAACATTGTTTAT |
| *Slc22a15*-3 | TCTACCTGATTAACCAAAGAT |
| *Slc22a15*-4 | GCCTGTCTTATTGTGATGTTT |
| *Slc22a15*-5 | CTGGCCTCATAGAGATTCCAT |
| *Bzw2*-1 | AGGTGGTGCTTTACGTCAAAG |
| *Bzw2*-2 | GCGATACTGAAGTGGTATAAA |
| *Bzw2*-3 | CCTGGGATGTATGCTTGTTAT |
| *Bzw2*-4 | CTGGGATGTATGCTTGTTATA |
| *Bzw2*-5 | ACCTTGAAGCTGTAGCCAAAT |
| *Slc41a2*-1 | CCGCGCTTCTTGGTCTTAAAG |
| *Slc41a2*-2 | GTTCACCCTGACTCCGTAAAT |
| *Slc41a2*-3 | CTCCTGTCTTGAGACATATTA |
| *Slc41a2*-4 | CTGATAACTCTCGCCATATTA |
| *Slc41a2*-5 | CCAGTTACTTAACTCACAATA |
| *Sh3bgrl2*-1 | CTCTGCCACCTCAGATATTTA |
| *Sh3bgrl2*-2 | CCCAGTATTTACATTTCCATA |
| *Sh3bgrl2*-3 | GAAGGAAAGCAACACAGTCTT |
| *Sh3bgrl2*-4 | GAAGCCAACAAGATAGAGTTT |
| *Sh3bgrl2*-5 | GATGTGGTTCGGTTTCTGGAA |
| *Fam46c*-1 | CATTGCGCACCCTCCAATTAC |
| *Fam46c*-2 | AGTACGACTACCTCATGATTC |
| *Fam46c*-3 | ACTTTCCAACCTTGGAGATAA |
| *Fam46c*-4 | TTCTGCCAGAGGGCGTGAATA |
| *Fam46c*-5 | AGCTGAGTTCTCGGTAGATTC |
| *Sh2b2*-1 | CCACTCCCATTAGCAGCTATT |
| *Sh2b2*-2 | CGGCTGATATCACCCTAAGAA |
| *Sh2b2*-3 | GAGCAGAATACATCCTGGAAA |
| *Sh2b2*-4 | GTGGAGAATCAGTACTCCTTT |
| *Sh2b2*-5 | GATCGGCTGATATCACCCTAA |
| *Gm5465*-1 | ACCACCTTAGCTGTCTATATT |
| *Gm5465*-2 | GGCCATTAGTGGCTCAATTAC |
| *Gm5465-*3 | GTTAGCACTCTAGTTACTTAA |
| *Gm5465*-4 | GGAGAGGTGTATGGGTATATA |
| *Gm5465*-5 | GCTACTGAGCAAGCCTATTGT |
| *Sema7a*-1 | GTGAACATCGGCTCCACAAAG |
| *Sema7a*-2 | ACTCAGCTGTCTGCGTGTATT |
| *Sema7a*-3 | CCATAGCTTTGTCTTCAATAT |
| *Sema7a*-4 | CTAAGTACCATTACCAGAAAG |
| *Sema7a*-5 | GCCATCCAGGCTATATCATTG |
| *Osbpl3*-1 | CCTGTAACTTTGCCCACATTT |
| *Osbpl3*-2 | CCCGGAAGCAATTTGTCATTT |
| *Osbpl3*-3 | CTCGGTTTAGACCAGATCAAA |
| *Osbpl3*-4 | CCTCAGTAATGATTTAGACAA |
| *Osbpl3*-5 | CCACAAAGTTTACTTCGCTTT |
| *Kpna2*-1 | GTGCTATCCCAGCGTTTATTT |
| *Kpna2*-2 | CGTGGGCTATAACCAACTATA |
| *Kpna2*-3 | TACTCAAGCTGCTCGGAAATT |
| *Kpna2*-4 | TCCTCTCTAAGGCGGACTTTA |
| *Kpna2*-5 | CCGACTTAACAGGTTCAAGAA |
| *Hpse*-1 | CGGATGGATTACTTTCCAAAT |
| *Hpse*-*2* | AGGCAAATAAGTGGAGGATAT |
| *Hpse*-3 | GCCGGATGGATTACTTTCCAA |
| *Hpse*-4 | CCACGATATCAGGAAGGAGAT |
| *Hpse*-5 | CCTTGACTACTGCTCTTCCAA |
| *Vwa3b*-1 | GCTTGGTCCACGTCAACATTA |
| *Vwa3b*-2 | GCTTCAGTTGCATGGGCTAAA |
| *Vwa3b*-3 | CAGCAATAAGATGGTATTAAT |
| *Vwa3b*-4 | GCAAGGGTATCCAACGAAGAA |
| *Vwa3b*-5 | CCCGCCATTGTTATAGCACTT |
| *Troap*-1 | GGCTCAACGGATACCATTAAA |
| *Troap*-2 | TAAGGATACAGCGCATCAATA |
| *Troap*-3 | TTTCGATATGACTGAACTAAA |
| *Troap*-4 | AGATTGCTATGAAGTTGTTTG |
| *Troap*-5 | ATTCGACGGAAAGCCCAATTC |
| *Brca1*-1 | CCACAGGTAAATCAGGAATTT |
| *Brca1*-2 | CCAAGAAGAGGATAGTATAAT |
| *Brca1*-3 | GTGCTTCCACACCCTACTTAC |
| *Brca1*-4 | CCCATCATACTTTAATGTGTA |
| *Brca1*-5 | CCTTTGTGTAAGAATGAGATA |
| *Hbb-bh1*-1 | GGGAAGGCTCCTGATTGTTTA |
| *Hbb-bh1*-2 | GATTGTCCTTTCTACTCATTT |
| *Hbb-bh1*-3 | TTAGAGCCCATGGCAAGAAAG |
| *Hbb-bh1*-4 | ACAACCTCAAGGAGACCTTTG |
| *Hbb-bh1*-5 | CAGAGATTCTTTGACAAGTTT |
| *Ccrl1*-1 | GCTGTAGCAGACTTGTTACTT |
| *Ccrl1*-2 | CAGTACGAAGTGATCTGCATA |
| *Ccrl1*-3 | CACACACACACACACACACAA |
| *Ccrl1*-4 | CTGCGATATGAGCAAACGCAT |
| *Ccrl1*-5 | GCATTGACAGATATTGGGCAA |
| *Lmo7*-1 | ATAATCATTGCCTCGTTAAAT |
| *Lmo7*-2 | TAGCAGGTTTGGATAACATAA |
| *Lmo7*-3 | ATGCCGTGGAACGAGATATAA |
| *Lmo7*-4 | ACTGCTATCTCCGATTCAAAT |
| *Lmo7*-5 | GTACCGAAGGCCCATTGATTC |
| *Bard1*-1 | ACAACGGCCATTGTGATATTT |
| *Bard1*-2 | CCACGGGTAAATGGTGAAATA |
| *Bard1*-3 | CGAAGTAAGAAGGTTAGATAT |
| *Bard1*-4 | GTTAAATCCACGTGGTGTATT |
| *Bard1*-5 | GCTTCTATTAAGGGTGATATA |
| *Ildr1*-1 | CGAGCAGATCTTGTGATTAAT |
| *Ildr1*-2 | TAATGGTGTCCTGGAATATTT |
| *Ildr1*-3 | AGGAAACCTATGGAATATTAT |
| *Ildr1*-4 | GCAGTATTGCTGCTGCTATAT |
| *Ildr1*-5 | GCTAATGGTGTCCTGGAATAT |
| *Optn*-1 | GAAGTCACAAAGAGGAATCTA |
| *Optn*-2 | GCCATGAAGCTAAATAATCAA |
| *Optn*-3 | CTGAAAGAGAACAATGACATT |
| *Optn*-4 | GAGCTGATGAAGAAGAGACTT |
| *Optn*-5 | GATAGGGACAAGCTCAGGATT |
| *Pdzd2*-1 | CTAGAGTCTGTTGAAGAATAT |
| *Pdzd2*-2 | GCTGAAGAAACAGCCAGATTT |
| *Pdzd2*-3 | TGCGCCATGCAGCTTTAAGTA |
| *Pdzd2*-4 | CCTGGAAGTAAATTCTGTGAA |
| *Pdzd2*-5 | GCAATGACAAACGCCGTTTCT |
| *Fads2*-1 | TCCACAAGGACCCGGACATAA |
| *Fads2*-2 | CCCACATCATCGTCATGGAAA |
| *Fads2*-3 | GCGTTTCTTCTACACCTACAT |
| *Fads2*-4 | GAAGCTGAAATACCTGCCCTA |
| *Fads2*-5 | CACCTTTCTGTCTATAAGAAA |
| *Lmnb1*-1 | GCGAATCTGATGGCCTTAATT |
| *Lmnb1*-2 | CCCAGCTAGAAGCATCCTTAT |
| *Lmnb1*-3 | GCGTCAGATTGAGTATGAGTA |
| *Lmnb1*-4 | GCTGCTCAATTATGCCAAGAA |
| *Lmnb1*-5 | CCCAGATCAAGCTTCGAGAAT |
| *Arhgap8*-1 | GCCCTGTTACCAAAGAATTTA |
| *Arhgap8*-2 | CCCGTGAACTTTGATGATTAT |
| *Arhgap8*-3 | GCCGTGATCCTGAAGACATTT |
| *Arhgap8*-4 | CCCTCATCAGTCACAAGTTTG |
| *Arhgap8*-5 | CGACTATACTATTGTCTACTT |
| *Endou*-1 | GATAAGACCACCTACGGAAAT |
| *Endou*-2 | GCCTACCTATGCAGCCTTTAT |
| *Endou*-3 | ACAAGAGTTTGTGGACGATTT |
| *Endou*-4 | CAAGAGTTTGTGGACGATTTG |
| *Endou*-5 | GCTGCCGTAGTTTGAAACATT |
| *Tmem48*-1 | GGCCGTGGTCATTATAATAAT |
| *Tmem48*-2 | ATCTCCCGTTTCCTATTATAC |
| *Tmem48*-3 | CCCACAATTGGACTGCTATTT |
| *Tmem48*-4 | GGTCTCCTGAACGTCTCATTA |
| *Tmem48*-5 | CAGATGCCCAAATGCATATTT |
| *Gen1*-1 | AGTATGGCTAATGCCTATAAT |
| *Gen1*-2 | TCCCAGAACTTTGGCTATAAA |
| *Gen1*-3 | TGAACGTAATGGATGCATATT |
| *Gen1*-4 | ATGTTGACTGTTACACGATAT |
| *Gen1*-5 | GGCATCAGACAGACCATAATA |
| *Ada*-1 | AGCCTATGAGGGCGCAGTAAA |
| *Ada*-2 | AGCCTCATCCTGTGGATAAAG |
| *Ada*-3 | CAAGCCAGAAACCATCTTATA |
| *Ada*-4 | GCGGTTGTTCGCTTCAAGAAT |
| *Ada*-5 | CGAGGATGAAGCTCTCTACAA |
| *Basp1*-1 | GCACCTGTAGTTCTGTTTATT |
| *Basp1*-2 | ACGAGAAGGCCAAGGACAAAG |
| *Basp1*-3 | CTACAATGTGAACGACGAGAA |
| *Basp1*-4 | GCCAAGGACAAAGACAAGAAG |
| *Basp1*-5 | GAAGGCCAAGGACAAAGACAA |
| *Phf19*-1 | CCTAGCCAGTATATTCGACTT |
| *Phf19*-2 | GCTCTCTATAACTTGGGAGTA |
| *Phf19*-3 | CCTCAAGTCCTCTATCACCAA |
| *Phf19*-4 | CGGGCTATATTACCTTGGCAA |
| *Phf19*-5 | CCCTTCAGAAAGGAAGGCTTT |
| *S1pr2*-1 | CCTCTACAAAGCCCACTATTT |
| *S1pr2*-2 | ACCGACATTTCTGGAGGGTAA |
| *S1pr2*-3 | GCCATCGTGGTGGAGAATCTT |
| *S1pr2*-4 | CACCCTTAACTCACTGCTCAA |
| *S1pr2*-5 | GCCTCTCTATGCTAAGCACTA |
| *Hmgb2*-1 | GCTACAACTACAGTTAGATTT |
| *Hmgb2*-2 | AGAGCGACAAAGCTCGTTATG |
| *Hmgb2*-3 | TTGGAGATACTGCGAAGAAAC |
| *Hmgb2*-4 | ATGTCCTCGTACGCCTTCTTC |
| *Hmgb2*-5 | CTGAGCAATCTGCCAAAGATA |
| *Depdc1a*-1 | AGTTTGGAGATACGTTATTAT |
| *Depdc1a*-2 | CCAAGTAATTCCTCAATATAT |
| *Depdc1a*-3 | CCATAGAACTTTCAGAGAAAT |
| *Depdc1a*-4 | CCTGCAACTTCTCCACTTAAA |
| *Depdc1a*-5 | CGTCTGTGTCTATATGTATAT |
| *Hmces*-1 | CAATGTCAGCTGACCTGAAAG |
| *Hmces*-2 | TATCACCTTCCATCCAGTTTC |
| *Hmces*-3 | TGACAATGGCAGGGATCTTTG |
| *Hmces*-4 | CTACCAACTGTCGTAGTGATA |
| *Hmces*-5 | CATAATGGAGAAGCAGTCATT |
| *Bmpr1a*-1 | GGGTCGTTACAACCGTGATTT |
| *Bmpr1a*-2 | CAATTTGTGCAACCAGTATTT |
| *Bmpr1a*-3 | GCTGGATGAAAGCCTGAATAA |
| *Bmpr1a*-4 | TCAAGACTCCAATCCTGATAA |
| *Bmpr1a*-5 | GCCCTACTCAAGTTAGCTTAT |
| *Ccnf*-1 | CCTACCGTGGTTGACTATAAA |
| *Ccnf*-2 | GCGACACCATGAGGTACATTC |
| *Ccnf*-3 | GCTCACAGACAACACGTATAA |
| *Ccnf*-4 | TGCTTGGGAAGGGTGCTAAAT |
| *Ccnf*-5 | CCTACTAAACGAAGAATCAAA |
| *Arhgap19*-1 | CAGCAACATCACAAGGCTTAT |
| *Arhgap19*-2 | GCTCCAGAACTCAAATGTCAA |
| *Arhgap19*-3 | GCTGATTGAATATCTGCACAA |
| *Arhgap19*-4 | CCAACTGGTATTTGAGGCTTT |
| *Arhgap19*-5 | CTTTATTACTTCTGTCCTCTA |
| *Aurkb*-1 | CTTCGCCGAGAGATCGAAATC |
| *Aurkb*-2 | CCAGCAGAGGATCTACTTAAT |
| *Aurkb*-3 | TGCCACAAGAAGAAGGTAATT |
| *Aurkb*-4 | CGCATGCATAATGAAATGGTA |
| *Aurkb*-5 | GCAGCCTTTCACTATTGACAA |
| *Hmgn3*-1 | CATCCTCAAGTCACGCAAATA |
| *Hmgn3*-2 | GATGGAACCAAGCTAACTAAG |
| *Hmgn3*-3 | CACAGAGAACTGAATCTATAG |
| *Hmgn3*-4 | CGCGAAACCTGTTCCACCAAA |
| *Hmgn3*-5 | AGAACCTGGAACAAAGATTAG |
| *Fignl1*-1 | GTTCGACCAATAGCTTATATT |
| *Fignl1*-2 | CTTGACTACTGACTGATATAT |
| *Fignl1*-3 | CCAGTACATTGGGACGATATT |
| *Fignl1*-4 | GTTATCTAGTAGTCGTCTTTA |
| *Fignl1*-5 | AGCCAGGAAACAGATAGTAAT |
| *Plxnb2*-1 | GCGTCTTTCTCCTGAGCAATA |
| *Plxnb2*-2 | CCAGTAGACATCAATAAGAAA |
| *Plxnb2*-3 | CCCAGCAACAAGCTGTTGTAT |
| *Plxnb2*-4 | CCAGGACATGAATACGCACTT |
| *Plxnb2*-5 | CCTGCTTAATAGCAAGTCCTT |
| *Lcp2*-1 | CAGTAAGTTGAGTCAAGATAT |
| *Lcp2*-2 | CCCTTCATACTAGGAAAGAAA |
| *Lcp2*-3 | CCATCGTTTCTTATTTGGTTT |
| *Lcp2*-4 | CCCTTAGATGAAGAGTGGTAT |
| *Lcp2*-5 | GCTGTGAAGAAATACCACATT |
| *Ect2*-1 | GAAAGCGGCACAAGGTTATTG |
| *Ect2*-2 | GCCACAATCATTCAATTATTT |
| *Ect2*-3 | ACCAGACTGAGAGCAATTATG |
| *Ect2*-4 | GAGCAAGGAAATGATCATTAA |
| *Ect2*-5 | TCGATTGTTTGATGTACTTAA |
| *Asns*-1 | CCCAGAAGTTTCCCTTCAATA |
| *Asns*-2 | GCTCTGTTACAATGGTGAAAT |
| *Asns*-3 | CCCTTATTTGTGGCTCTGTTA |
| *Asns*-4 | CGTGAAGAACAATCTGCGTAT |
| *Asns*-5 | CGCTATCAAGAAACGCTTGAT |
| *Neil3*-1 | GCTGAAGATTGGACCTAATAA |
| *Neil3*-2 | CCCGTCTCATAAACTCATCAA |
| *Neil3*-3 | CTTGAGACTGTTTAATGGATA |
| *Neil3*-4 | TCGGGATTTCAGCATTCTCTT |
| *Neil3*-5 | GCACTCTTTGACAGTGGTCTT |
| *Eaf2*-1 | GATGTCTCCAACGTCTCTAAT |
| *Eaf2*-2 | CCTATCTGATATGCCTTAAAT |
| *Eaf2*-3 | CATCTTCAAGTAGTGAGGATA |
| *Eaf2*-4 | GCAAGATAGATGTCAGTGTTA |
| *Eaf2*-5 | GATGCTACTTGTCACCGACTT |
| *Dhfr*-1 | GTAAACAGAATCTGGTGATTA |
| *Dhfr*-2 | CCTGAGAAGAATCGACCTTTA |
| *Dhfr*-3 | GTTTGAAGTCTACGAGAAGAA |
| *Dhfr*-4 | GACTTATTGAACAACCGGAAT |
| *Dhfr*-5 | CTGGTGATTATGGGTAGGAAA |
| *Car13*-1 | GCAGTCTCCAATTGAGATTAA |
| *Car13*-2 | TTGACGACACGGAGGACAAAT |
| *Car13*-3 | GACCTCTCAGTATCAAGTATG |
| *Car13*-4 | GTATGTCCAATTGCTAAATAT |
| *Car13*-5 | TGGCTGTCCTGGGAGTATTTC |
| *Hmmr*-1 | ACTGCCTAGTCTTAGGTATAT |
| *Hmmr*-2 | GACTCTCAGAAGAATGATAAA |
| *Hmmr*-3 | CGAGCTACTAAAGGCTAAGTT |
| *Hmmr*-4 | GCCAGCTACTTGAAACAGAAA |
| *Hmmr*-5 | CAGGCATTGTTGAATGAACAT |
| *Anxa2*-1 | GTATGATGCTTCGGAACTAAA |
| *Anxa2*-2 | AGTTATTGACTACGAGCTGAT |
| *Anxa2*-3 | CGAGACAAGGTCCTGATTAGA |
| *Anxa2*-4 | GTTATTGACTACGAGCTGATT |
| *Anxa2*-5 | GAGCATCAAGAAAGAGGTCAA |
| *Rassf6*-1 | CCCTCGGGATTTCGCTCTTTA |
| *Rassf6*-2 | CGGATCTTCCTCATGGATAAA |
| *Rassf6*-3 | CATGAGAACTGAAGAAGTAAT |
| *Rassf6*-4 | CAGAGTAAACAGGCAGCTAAA |
| *Rassf6*-5 | TGACCATGAGACATCCATCTT |
| *Gcet2*-1 | CCCGTTCAGGACAATGCTAAT |
| *Gcet2*-2 | GAAGAGTTGTGCTACATCCTT |
| *Gcet2*-3 | CTGAGTATTCGGTTCTCCGTT |
| *Gcet2*-4 | CCCACGTCAATCATAAATCAA |
| *Gcet2*-5 | CTGAGAGACACAAAGAGTCTT |
| *Gm600*-1 | CGGACTCAAGTATCTCATAAA |
| *Gm600*-2 | GCAGCATCACTACCATGTTAA |
| *Gm600*-3 | GATCAAATATGTGGAAGTGAT |
| *Gm600*-4 | CCAGTTCATTCCAAGGCGTTT |
| *Gm600*-5 | GCTCTGATGCAGCACATAGAT |
| *Top2a*-1 | CCTCTCTAATAACAGACTATA |
| *Top2a*-2 | GCAGACTACATTGCCGTTTAA |
| *Top2a*-3 | GCTCGCTTTATATTAGAGAAA |
| *Top2a*-4 | CCCGAGTTTGAAGAATGGAAA |
| *Top2a*-5 | CCAGCAGATTAGCTTCGTCAA |
| *Mybl1*-1 | GGATTCAGAACTCCTACTATT |
| *Mybl1*-2 | CCCTTGTAAAGCTGTCAAATT |
| *Mybl1*-3 | CCAAACCCTAACCCTTGTAAA |
| *Mybl1*-4 | AGATCCCTGGGTATCAGTATG |
| *Mybl1*-5 | CCCACAAAGTTCCTAGCTGTA |
| *Melk*-1 | ACGCAGAGCAGTGGCAAATAA |
| *Melk*-2 | AGTTAGTAAGAACCAGTATAA |
| *Melk*-3 | CTGGAGAGATGGTAGCTATAA |
| *Melk*-4 | CTGTTAAGTAGAGACTATTTG |
| *Melk*-5 | GCCTGGGTTTACAAGAGATTA |
| *Shcbp1*-1 | GATCTGTTGTCTGGTATAAAT |
| *Shcbp1*-2 | TCGAGTTCCCTCGGGACTTAT |
| *Shcbp1*-3 | CGATGATAAATAACGTCATAC |
| *Shcbp1*-4 | TCCATGGTGGAAGGGTTAAAT |
| *Shcbp1*-5 | TCTTAAGTATGCTGCTTATAT |
| *Zdhhc2*-1 | GCATTCAGAAATCCAGTATTT |
| *Zdhhc2*-2 | GTGACAGATGCCAACTTATAA |
| *Zdhhc2*-3 | CTCAAGCCAAGTTCCATATTA |
| *Zdhhc2*-4 | CTACTCCTGCGGGACTAAATT |
| *Zdhhc2*-5 | GCATTGACTATGAACTCATTA |
| *Sqle*-1 | GAGCCCAATGTAAAGTTTATA |
| *Sqle*-2 | GCTTGTGTATAAGCATGTAAA |
| *Sqle*-3 | CCAGTTCTCATCTACCAGATT |
| *Sqle*-4 | CCATCATATACATGGCTACAT |
| *Sqle*-5 | GCCACGTATTTCTGCTTCAAA |
| *Ube2j1*-1 | GCTCTTATATTCCGACGAATA |
| *Ube2j1*-2 | ATCGGGTTTATGCCGACTAAA |
| *Ube2j1*-3 | CCATGAAACCACCAAGCATTA |
| *Ube2j1*-4 | ACATTCTGCATTGGGTATAAT |
| *Ube2j1*-5 | CGACGAATATATCTGGCCAAT |
| *Bcat1*-1 | CCCAGCACATAGTAGGTATTT |
| *Bcat1*-2 | GATGGGAGAAACCTCACATTA |
| *Bcat1*-3 | CGGACCTCAACATGGATAGAA |
| *Bcat1*-4 | CCTTCCAAAGCCCTACTCTTT |
| *Bcat1*-5 | GCATATTCCAACGATGGAGAA |
| *Rrm2b*-1 | CTCACTGGAACAAGCTTAAAT |
| *Rrm2b*-2 | CATGGCCGAAACCACAGATAA |
| *Rrm2b*-3 | CTGGATCATTTGCTGCAATAT |
| *Rrm2b*-4 | GAAACTTTCAGTATCTCATTT |
| *Rrm2b*-5 | GAGATGTACAGTTTACTAATA |
| *Rrm2*-1 | GCGAGTGATGGCATAGTCAAT |
| *Rrm2*-2 | CGTTGTCTTTCCCATCGAGTA |
| *Rrm2*-3 | CTCCTTATTGACACTTACATT |
| *Rrm2*-4 | CCATGATATCTGGCAGATGTA |
| *Rrm2*-5 | TCTCACTAGAAGGAAAGACAA |
| *Tspan32*-1 | TCTATGGCATCAGGAACATAA |
| *Tspan32*-2 | ATGTTGAGGTCAACCAATTTC |
| *Tspan32*-3 | CTGTGGACACACTACAGTATT |
| *Tspan32*-4 | GATCCTCAAAGGAGTAGATAA |
| *Tspan32*-5 | GACACCTATGACTTCGTGTAT |
| *Gpr174*-1 | CTAGCCATTGCTGACTTATTA |
| *Gpr174*-2 | CTGTCTTGACCCAGTTATATA |
| *Gpr174*-3 | ATATTAGCCTTATGGGTATTT |
| *Gpr174*-4 | TGATAATACTGCGCACATATT |
| *Gpr174*-5 | TGCATCAGTGTGCGAAGATTT |
| *Adgre5*-1 | GCGTCTGTAACCTGGGATATA |
| *Adgre5*-2 | GTCTGTAACCTGGGATATAAG |
| *Adgre5*-3 | GATTCCGAGTGTCTCACTTAA |
| *Adgre5*-4 | TCACGTTCAAGTTCGACTTTA |
| *Adgre5*-5 | ATGAAGGACAGCAGCACATAC |
| *Cd200*-1 | TACTGGAAACGTCACCGAAAT |
| *Cd200*-2 | GTCTGTTGTAGGACTTGATTT |
| *Cd200*-3 | TAATCCAGCCTGCCTACAAAG |
| *Cd200*-4 | CAGAGTCTGGACAAAGGATTT |
| *Cd200*-5 | GCATCCTTACGATGTTCTCTA |

**Supplementary Table 2.** List of shRNAs recovered in non-GC or GC B cells. “Log_2_(fold change) (GC/non-GC)” is calculated as the GC/non-GC ratio for the abundance of each shRNA construct, expressed as binary logarithm (log2); “SD^-1^” is calculated as the reciprocal of SD.

| ShRNA | Log_2_(fold change) (GC/non-GC) | | Mean | SD^-1^ | Mean^2^SD^-1^ |
| --- | --- | --- | --- | --- | --- |
| *Slc43a3*-3 | -5.7 | -5.5 | -5.60 | 7.07 | 302.2 |
| *Ccrl1*-5 | 6.5 | 6.1 | 6.33 | 3.54 | 133.5 |
| *Fut8*-3 | -5.9 | -5.4 | -5.66 | 2.83 | 80.6 |
| *Zdhhc2*-2 | -7.1 | -6.0 | -6.53 | 1.29 | 57.2 |
| *Mybl1*-5 | -5.9 | -7.2 | -6.56 | 1.09 | 49.1 |
| *Melk*-2 | 8.0 | 6.2 | 7.10 | 0.79 | 40.3 |
| *Fignl1*-3 | -8.5 | -6.2 | -7.38 | 0.61 | 32.9 |
| *Adgre5*-1 | 7.0 | 10.6 | 8.78 | 0.39 | 30.0 |
| *Shcbp1*-1 | -4.3 | -6.0 | -5.17 | 0.83 | 22.1 |
| *Sh2b2*-4 | 4.8 | 7.9 | 6.35 | 0.46 | 18.9 |
| *Smco4*-5 | -5.2 | -2.5 | -3.84 | 0.52 | 7.8 |
| *Akr1e1*-1 | -1.3 | -1.7 | -1.46 | 3.54 | 7.3 |
| *Hmgb2*-1 | 2.3 | 8.4 | 5.31 | 0.23 | 6.6 |
| *Shcbp1*-4 | 1.3 | 1.0 | 1.15 | 4.71 | 5.8 |
| *Top2a*-3 | 5.3 | 2.0 | 3.64 | 0.43 | 5.7 |
| *Pask*-4 | 7.1 | 1.8 | 4.41 | 0.27 | 5.2 |
| *Arhgap8*-4 | 6.3 | 1.7 | 3.95 | 0.31 | 4.8 |
| *Lmo7*-2 | 5.0 | 1.7 | 3.34 | 0.43 | 4.8 |
| *Rrm2b*-1 | -7.9 | -1.1 | -4.50 | 0.21 | 4.2 |
| *Akr1e1*-2 | -1.3 | -6.2 | -3.74 | 0.29 | 4.1 |
| *Lcp2*-2 | -6.0 | -1.2 | -3.59 | 0.29 | 3.8 |
| *Gen1*-1 | 0.9 | 6.9 | 3.91 | 0.24 | 3.6 |
| *Rrm2b*-5 | -0.9 | -6.7 | -3.80 | 0.24 | 3.5 |
| *Gpr174*-1 | 8.3 | 0.4 | 4.32 | 0.18 | 3.3 |
| *Rrm2b*-4 | 5.4 | 0.9 | 3.17 | 0.31 | 3.1 |
| *Hmmr*-2 | -6.0 | -0.7 | -3.34 | 0.27 | 3.0 |
| *Hbb-bh1*-5 | -5.6 | -0.7 | -3.15 | 0.29 | 2.9 |
| *Plxnb2*-2 | -0.8 | -5.1 | -2.96 | 0.33 | 2.8 |
| *Troap*-1 | 5.5 | 0.7 | 3.10 | 0.29 | 2.8 |
| *Bmpr1a*-5 | 7.0 | 0.3 | 3.62 | 0.21 | 2.8 |
| *Cd200*-5 | -0.8 | -1.9 | -1.35 | 1.29 | 2.5 |
| *Zdhhc2*-5 | -5.9 | -0.2 | -3.05 | 0.25 | 2.3 |
| *Tmem48*-2 | -5.8 | -0.1 | -2.94 | 0.25 | 2.2 |
| *Slc22a15*-2 | -2.7 | -0.7 | -1.73 | 0.71 | 2.1 |
| *Depdc1a*-5 | -5.5 | -0.1 | -2.80 | 0.26 | 2.1 |
| *Lcp2*-3 | -5.1 | -0.2 | -2.65 | 0.29 | 2.0 |
| *Adgre5*-4 | 4.5 | 0.3 | 2.38 | 0.34 | 1.9 |
| *Melk*-5 | -4.5 | -0.3 | -2.38 | 0.34 | 1.9 |
| *Ect2*-5 | -6.2 | 0.3 | -2.93 | 0.22 | 1.9 |
| *Rassf6*-3 | -5.0 | 0.0 | -2.51 | 0.28 | 1.8 |
| *Optn*-4 | -7.7 | 1.2 | -3.25 | 0.16 | 1.7 |
| *Neil3*-5 | 6.9 | -0.8 | 3.02 | 0.18 | 1.7 |
| *Vwa3b*-3 | 0.5 | -5.6 | -2.57 | 0.23 | 1.5 |
| *Depdc1a*-2 | 5.7 | -0.6 | 2.54 | 0.22 | 1.4 |
| *Arhgap11a*-1 | 1.1 | -6.6 | -2.75 | 0.18 | 1.4 |
| *Eaf2*-4 | 0.5 | 1.2 | 0.82 | 2.02 | 1.4 |
| *Fignl1*-1 | -4.8 | 0.5 | -2.16 | 0.27 | 1.2 |
| *Arhgap8*-1 | -0.3 | -1.6 | -0.94 | 1.09 | 1.0 |
| *Hbb-bh1*-2 | -4.4 | 0.7 | -1.87 | 0.28 | 1.0 |
| *Lmnb1*-4 | -3.2 | 8.8 | 2.81 | 0.12 | 0.9 |
| *Fignl1*-2 | 3.6 | -0.4 | 1.60 | 0.35 | 0.9 |
| *Akr1e1*-4 | 6.4 | -1.9 | 2.27 | 0.17 | 0.9 |
| *Bard1*-3 | 0.1 | -2.5 | -1.21 | 0.54 | 0.8 |
| *Slc22a15*-3 | 0.6 | 0.3 | 0.41 | 4.71 | 0.8 |
| *Asns*-2 | -0.5 | -0.2 | -0.35 | 4.71 | 0.6 |
| *Tspan32*-4 | -4.8 | 1.5 | -1.68 | 0.22 | 0.6 |
| *Car13*-4 | -0.6 | -0.2 | -0.36 | 3.54 | 0.5 |
| *Ildr1*-3 | 0.1 | -1.2 | -0.59 | 1.09 | 0.4 |
| *Brca1*-5 | 0.3 | -1.7 | -0.69 | 0.71 | 0.3 |
| *Aurkb*-5 | -0.1 | 0.9 | 0.41 | 1.41 | 0.3 |
| *Rrm2*-5 | -5.3 | 8.4 | 1.56 | 0.10 | 0.3 |
| *Smco4*-3 | -5.5 | 3.1 | -1.18 | 0.16 | 0.2 |
| *Smco4*-1 | 4.0 | -6.5 | -1.27 | 0.13 | 0.2 |
| *Slc41a2*-3 | 5.1 | -7.8 | -1.37 | 0.11 | 0.2 |
| *Car13*-1 | 0.1 | -0.9 | -0.37 | 1.41 | 0.2 |
| *Ect2*-2 | 1.1 | -0.3 | 0.44 | 1.01 | 0.2 |
| *Fads2*-5 | 0.0 | -0.5 | -0.23 | 2.83 | 0.2 |
| *Mybl1*-1 | -6.1 | 8.7 | 1.31 | 0.10 | 0.2 |
| *Eaf2*-3 | -6.3 | 8.6 | 1.15 | 0.09 | 0.1 |
| *Tspan32*-5 | 1.5 | -0.7 | 0.41 | 0.64 | 0.1 |
| *Depdc1a*-1 | 6.4 | -8.4 | -0.99 | 0.10 | 0.1 |
| *Hpse*-4 | 6.1 | -7.8 | -0.84 | 0.10 | 0.1 |
| *Top2a*-1 | 0.6 | -0.2 | 0.20 | 1.77 | 0.1 |
| *Troap*-3 | -2.9 | 1.9 | -0.48 | 0.29 | 0.1 |
| *Bard1*-5 | -0.5 | 0.2 | -0.18 | 2.02 | 0.1 |
| *Dck*-2 | -5.1 | 6.4 | 0.66 | 0.12 | 0.1 |
| *Brca1*-2 | 0.1 | -0.3 | -0.11 | 3.54 | 0.0 |
| *Mybl1*-2 | -1.0 | 0.6 | -0.19 | 0.88 | 0.0 |
| *Lmo7*-3 | -1.0 | 1.4 | 0.21 | 0.59 | 0.0 |
| *Arhgap8*-5 | -0.9 | 0.6 | -0.16 | 0.94 | 0.0 |
| *Basp1*-1 | -2.3 | 2.8 | 0.27 | 0.28 | 0.0 |
| *Osbpl3*-4 | 4.6 | -5.1 | -0.26 | 0.15 | 0.0 |
| *Brca1*-1 | -6.0 | 6.6 | 0.30 | 0.11 | 0.0 |
| *Mybl1*-4 | -0.6 | 0.5 | -0.05 | 1.29 | 0.0 |
| *Optn*-2 | 0.3 | -0.4 | -0.03 | 2.02 | 0.0 |
